# Selpercatinib in Treatment of Advanced Medullary Thyroid Cancer: Effectiveness and Side Effects – A Case Report of 2 Patients during 60 Months of Therapy

**DOI:** 10.1159/cro/abzag009

**Published:** 2026-08-22

**Authors:** Luciano Severgnini, Bernd Hartmann, Doz. Thomas Winder, Theresia Lang, Andreas Volgger, Magdalena Benda, Patrick Reimann

**Affiliations:** a Institute of Internal Medicine II: Oncology and Hematology, Gastroenterology and Infective Disease, Landeskrankenhaus of Feldkirch, Feldkirch, Austria; b University in Principality of Liechtenstein (UFL), Triesen, Liechtenstein; c University and Oncological Center of Zurich, Swiss Tumor Immunology Institute, Zurich, Switzerland

**Keywords:** Thyroid carcinoma, Tyrosine kinase inhibitor, Metastasis, Selpercatinib, RET mutation

## Abstract

**Introduction:**

Medullary thyroid carcinoma (MTC) is a rare, but aggressive tumor that accounts for 4% of thyroid carcinomas. A mutation in the proto-oncogene RET has been shown to occur in ca. 70% of those neoplasia. Selpercatinib is a newly approved drug that specifically targets the RET-kinase and demonstrated a very high efficacy in reducing the tumor activity and in progression-free survival in patients with an advanced metastatic disease [1].

**Case Presentation:**

In accordance with the approval of the FDA, we started a treatment with selpercatinib on 2 patients with advanced metastatic MTC with an established RET mutation. They received selpercatinib 160 mg bidaily, and we observed the patients for 60 months. We observed a very good metabolic and serological response to the treatment with a decrease in the tumor marker of 99.8% and 98.5% and stable imaging. Hypertension grade II, hypotension grade I, proteinuria grade I, hyperphosphatemia grade I, and hypocalcemia grade II were the low-grade side effects that we observed during these 60 months of therapy.

**Conclusion:**

Selpercatinib proved to be a good antineoplastic drug for patients with RET-mutated, advanced, and inoperable MTC.

## Background

MTC is a rare, but aggressive tumor of the thyroid. It accounts for 4% of thyroid carcinomas and originates from parafollicular C cells, secreting calcitonin.

A mutation in the proto-oncogene RET that translates to produce a tyrosine kinase is a recurrent alteration in the oncogenesis and progression in ca. 70% of MTC and in ca. 10% of other non-medullary thyroid cancers. Here, 25% of total RET-mutated MTCs originate through germline mutations of RET (typical in hereditary multiple endocrine neoplasia MEN 2A and 2B) [1]. The remaining 75% of RET-mutated MTCs are somatic RET mutations. This mutation codes a ligand-independent signaling responsible for oncogenesis. The incidence of RET mutation is higher in thyroid cancer diagnosed in children and increases in cancers linked with environmental radiation exposure.

Therapies with multitargeted kinase inhibitors such as vandetanib or cabozantinib are already in use for the medullary thyroid carcinoma (MTC) with an objective response rate between 28 and 45% and progression-free survival of 4 to 17 months [2, 3]. They target RET together with other kinases, but they proved to have high toxic side effects due to their nonspecific target activity against multiple kinases. This can be seen especially through the inhibition of the activity of the receptor for the vascular endothelial grow factor which is part of the antineoplastic activity, but can increase the risk of bleeding and have a negative effect wound healing.

One of the newly developed RET tyrosine kinase inhibitors named selpercatinib (LOXO-292) showed a very promising and selective activity in the management of these RET-driven MTCs as well as other RET-altered malignancies [4–7]. In 2018, selpercatinib (LOXO-292) received US Food and Drug Administration Breakthrough Therapy designation for treatment of patients with previously treated metastatic RET-mutant MTC and others RET-altered malignancies [8].

## Methods

As a principal product of the C parafollicular cells of the thyroid, calcitonin is a hormone involved in the metabolism of calcium in the body. For this reason, calcitonin is the main product and the most sensitive and specific tumor marker of most medullary thyroid cancers. Another important tumor marker secreted by MTC is the carcinoembryonic antigen. We used calcitonin as a serological marker of response to therapy in our observation. The carcinoembryonic antigen was used in parallel to measure the response of therapy as part of the observations. Our laboratory performed the routine mensurations using a Cobas e801 analyzer (Roche Diagnostics).

We enrolled 2 patients with advanced metastatic neoplasia whose progress was monitored by a multidisciplinary team composed of oncologists, surgeons, radiologists, and nuclear medicine specialists of the LKH of Feldkirch (Austria). Molecular analysis was performed by high-resolution melting analysis of DNA, able to diagnose mutations in the exons 10, 11, 13, 14, and 15 in the *RET* gene.

The treatment with selpercatinib was initiated with 160 mg bidaily as established in the clinical trials [8]. We performed imaging at the point in time 0 before treatment start and after 12 months of treatment with a 68Ga-Peptide-SSTR PET-CT. In addition, the serological response was assessed by measurement of calcitonin at several predefined check-ups. We followed the serological response and observed the possible occurrence of side effects with initial biweekly and then monthly follow-up appointments.

## Results

### Case Presentation 1

Our Pat.1 (50 years old) had a first diagnosis of a nodular enlargement of the thyroid gland without alterations in the thyroid hormones in July 2007 in Moldova (at the time he was 35 years old). To proceed in the diagnosis, a thyroid scintigraphy was performed, where the nodular structure was cold. A fine-needle aspiration was performed, and the cytology revealed monomorphic spindle cells consistent with MTC.

In September 2007, a total thyroidectomy was performed; histologically, it was a low differentiated MTC (staging pT3a Nx M0). In October 2007, radioiodine therapy was performed (5,000 MBq [131I]). We do not have any data about the performance of molecular pathological or genetical test after the histological diagnosis as all of this treatment and diagnosing had been performed in Moldova.

The patient had no comorbidities at that time. In the postoperative course, the patient did not receive any follow-up care at home and between 2008 and 2020 the patient did not have any contact with doctors and did not undergo regular imaging.

In April 2020, the patient came to our attention for the first time. Due to a small intestine perforation of the terminal ileum, the patient was admitted to our visceral surgery and operated on. During the hospitalization, a sonography was performed in which multiple liver metastases were diagnosed. After a core-needle biopsy, the histology confirmed the diagnosis of MTC. At this time, a molecular pathological and genetical analysis was performed and the high-resolution melting analysis of DNA showed a mutation in the exon 14/codon 804 with a heterozygotic germline mutation of the *RET* gene c.1901G>A (MEN2A).

In a 68Ga-DOTATATE-PET/CT, multiple osseous and lymphogenic metastases as well as hotspots in the liver were detected (Fig. 1a–c). The basal calcitonin value was 105,375 pg/mL at the diagnosis of metastasis (Fig. 3 point A).

**Fig. 1.**
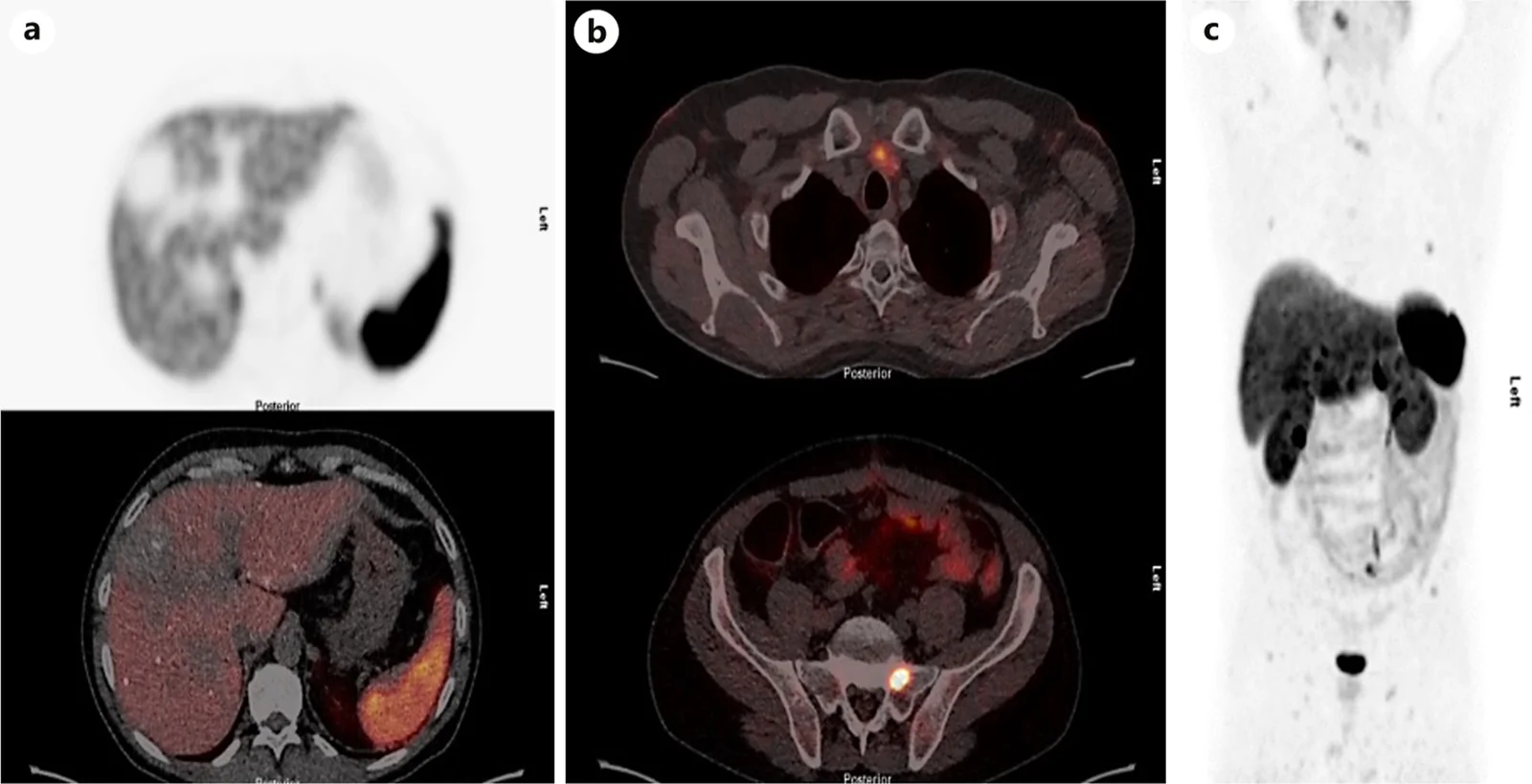
**a–c** Baseline 68Ga-DOTATATE-PET/CT*. **a** (Top and bottom) multiple calcified liver clusters without expression of somatostatin receptors, therefore without storage in 68Ga-DOTATATE-PET/CT. **b** (top) SSTR-positive calcified lymph metastasis paratracheal and (bottom) bone metastases in the sacrum region. **c** multi-intensity projection (MIP) showed multiple lymphatic metastases in the throat and bone metastases.

**Fig. 3.**
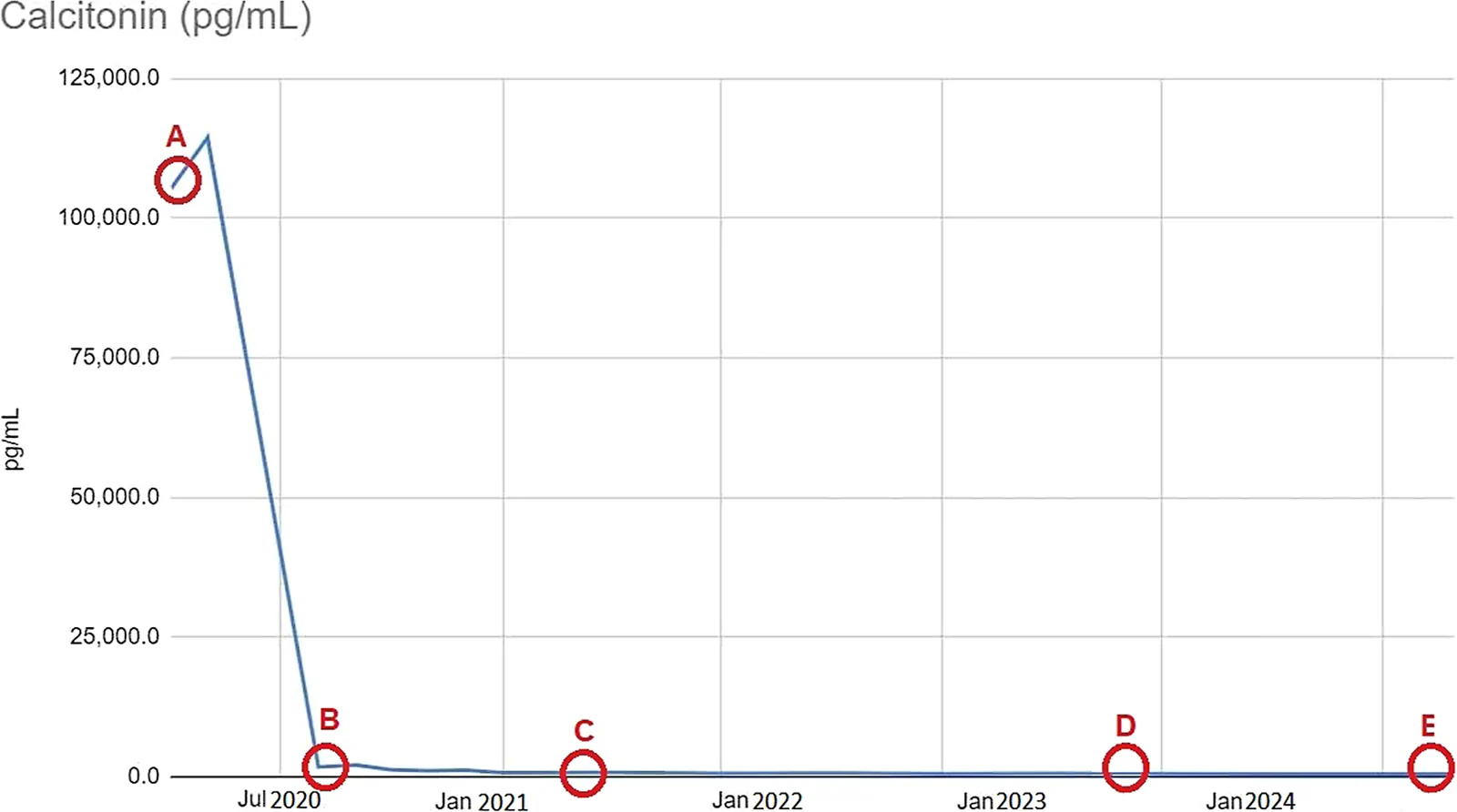
A-E: Evolution of calcitonin values during selpercatinib therapy from August 2020.

#### Therapy with Selpercatinib

In early August 2020, the patient was enrolled in the selpercatinib Named Patient Program as the first-line therapy in metastatic diffuse disease. Despite the metastatic situation, no additional radiation was necessary. The patient was prescribed selpercatinib 320 mg daily (160 mg twice daily). After 20 days, we observed a rapid decrease in the calcitonin value of more than 99% from 10,5375 pg/mL at diagnosis of metastasis (Fig. 3 point A) to 1,790 pg/mL under this targeted therapy (Fig. 3 point B).

After 1 year of therapy, we performed a 68Ga-Peptide-SSTR PET-CT (Fig. 2a, b) and a CT scan with which we observed stability in the dimensions of the tumor (RECIST 1.1: SD) mass and a reduction of the tumor metabolisms. Asymptomatic hyperphosphatemia grade II was documented 2 months after initiation of full-dose selpercatinib, and therefore, treatment with sevelamer 800 mg 3 times a day was initiated. Mild hypocalcemia had been known since the diagnosis but worsened to grade II upon initiation of the therapy. For this reason, the patient received supplementation with calcium carbonate. As part of these adjustments, the dose of selpercatinib was reduced to 240 mg daily.

**Fig. 2.**
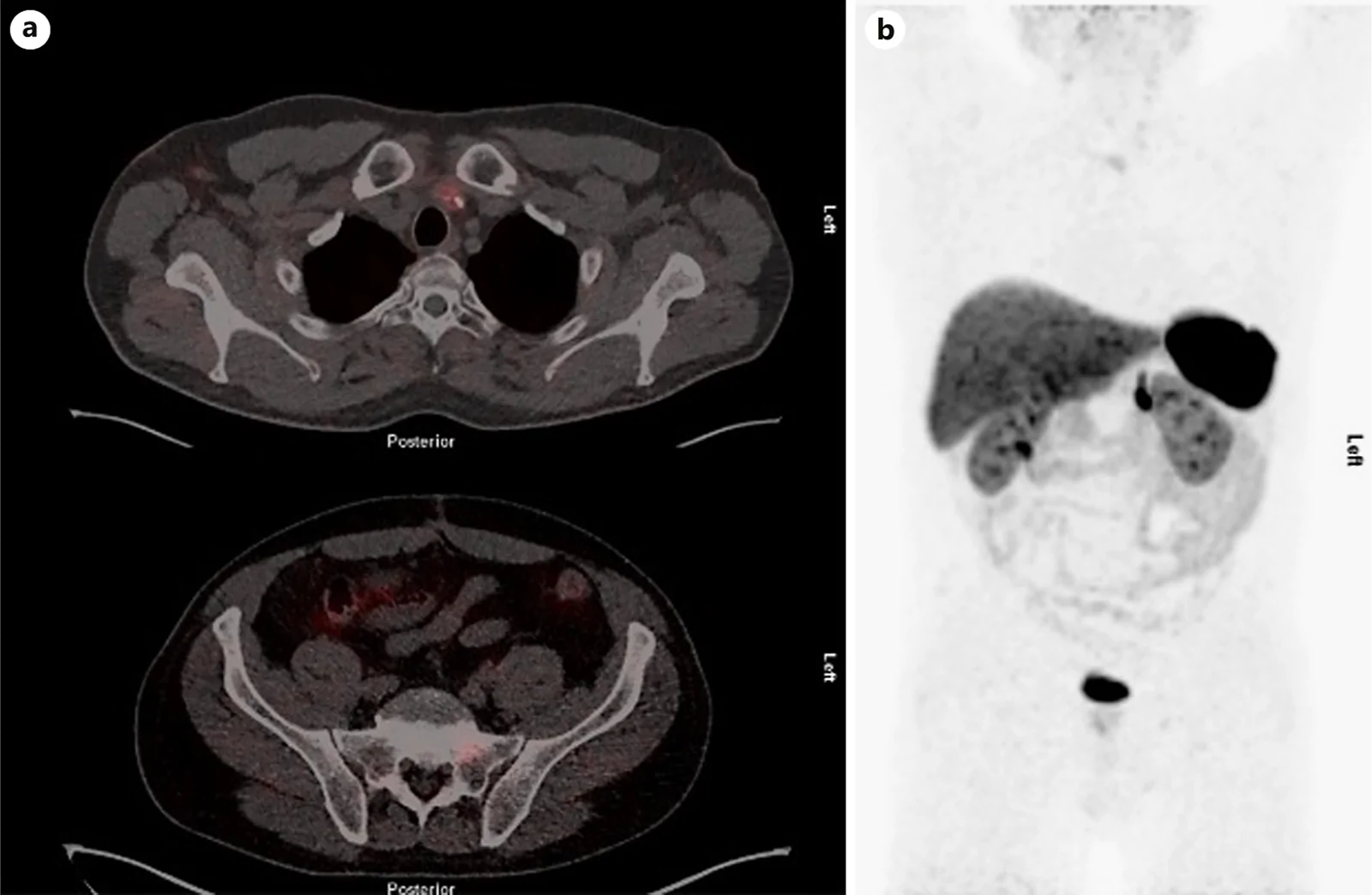
**a, b** Corresponding 68Ga-DOTATATE PET/CT* images after 1 year of treatment with selpercatinib showed a marked reduction in tracer binding in nodal metastases (**a** above example paratracheal) and osseous metastases (**a** below example sacrum). **b** The MIP where multiple lymphatic metastases in the neck and bone metastases are decreasing compared to **c**. MIP, multi-intensity projection.

After 6 months of therapy, in February 2021, we diagnosed arterial hypertension grade III (RR: 160/102 mmHg), so we started treatment with candesartan 16 mg initially as monotherapy and after observation combined with amlodipine 5 mg. Due to impaired phosphate and calcium balance, as well as treatment-resistant diastolic hypertension, the dose of selpercatinib was maintained to 240 mg daily.

At the follow-up visit in mid-March 2021, the patient showed a good general condition (ECOG 0). The calcitonin value was reduced again by >99% (624 pg/mL) and stable as such (Fig. 3 point C). The hyperphosphatemia completely returned to normal parameters, and the hypocalcemia was stable under supplementation. The blood pressure was better controlled with antihypertensives (RR: 138/104 mmHg), but the diastolic pressure was still increased (grade II) and the patient was still not completely compliant with the recommended therapy.

At the beginning of May 2022, the patient’s health status was stable (ECOG 0), calcitonin value: 502 pg/mL. Calcium, phosphate, and blood pressure were normal.

During the years 2023, 2004, and 2025, the general condition was good (ECOG 0). Calcitonin at the lowest point (185 pg/mL) 99.8% suppressed (August 2023, Fig. 3 point D) and so remained in our last control in August 2025 (Fig. 3 point E). The patient’s compliance with blood pressure therapy was not always good, which is why the values always tended to be elevated (hypertension grades I to II). Overall, however, the chronic therapy with selpercatinib was well tolerated for 5 years.

In this patient, the drug demonstrated good tolerability and sustained stable disease in PET-CT and stable partial remission of the tumor markers and sustained tumor suppression that persisted until the point in time this case report was written. The therapy is therefore currently being continued. The next clinical and laboratory chemical control examination is scheduled for December 2025.

### Case Presentation 2

Our Pat.2 (currently 76 years old) was first diagnosed with a struma nodosa in December 1988. In April 1997, a cold nodular lesion in the thyroid (47 mL) was biopsied, which was cytologically classified as follicular neoplasia with atypia. In November 1997, a total thyroidectomy was performed; histologically, it was confirmed as a mixed follicular thyroid carcinoma)/MTC (staging pT3 Nx M0). Radioiodine therapy (5,550 MBq [131I]) was performed in January 1998. Until 2008, we observed a complete remission of neoplasia. Since 2008, however, there have been suspicions of a relapse. In June 2009, a sonography revealed a thyroid carcinoma on the right (4.4 mL) of the original localization. The diagnosis was definitely confirmed by a [18F]-FDG-PET (Fig. 4).

**Fig. 4.**
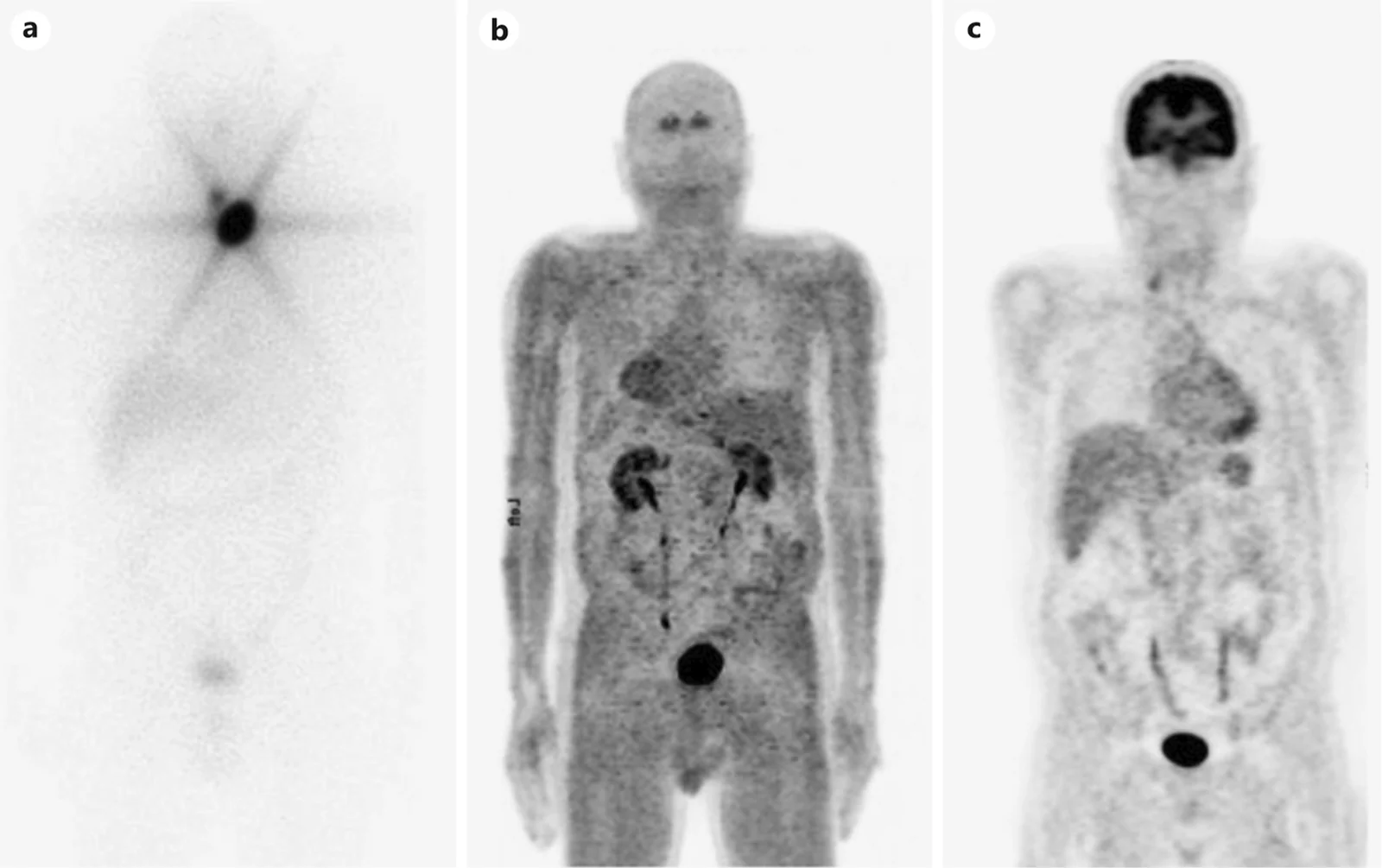
Scintigraphy (**a**), FDOPA PET-CT (**b**), and [18F]-FDG-PET-CT scan (**c**) at the time of relapse 2009.

At the time, the basal calcitonin level was 23.3 pg/mL (Fig. 8 point A). In July 2009, a central neck dissection was performed with removal of the lateral right neck lymph nodes (2 of 47 lymph nodes affected). A RET germline mutation (exon 14, codon 804 responsible for MEN2A syndrome) was detected by molecular genetics. It was therefore a familial MTC. In August 2009, the postoperative calcitonin level was 26.3 pg/mL that remained stable until January 2014. From October 2014 onward, however, a continuous increase in the basal calcitonin value over the years was observed (Fig. 8 point B). [18F]-Fluorodopa-PET/CT was negative in April 2015. In December 2015, a 68Ga-DOTATATE-PET/CT was performed in which multiple osseous metastases and pelvic lymph node metastases were presented (Fig. 5).

**Fig. 8.**
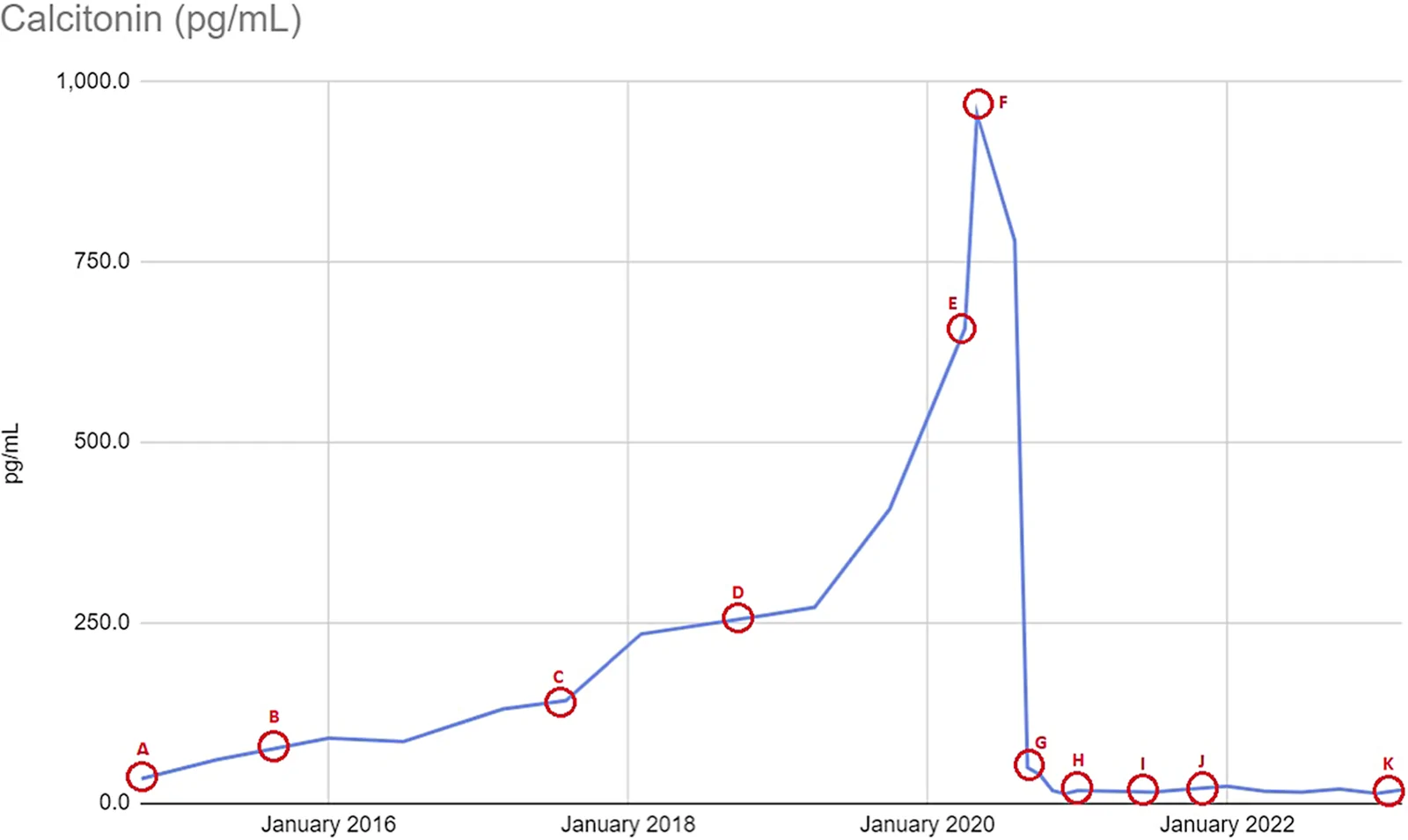
A to K: We can observe the long progression of the illness from the first documentation of altered calcitonin in October 2014 until beginning of the therapy in August 2020 (F to G). After that, the fall of the calcitonin level in blood and the ongoing maintenance of the remission status for the patient.

**Fig. 5.**
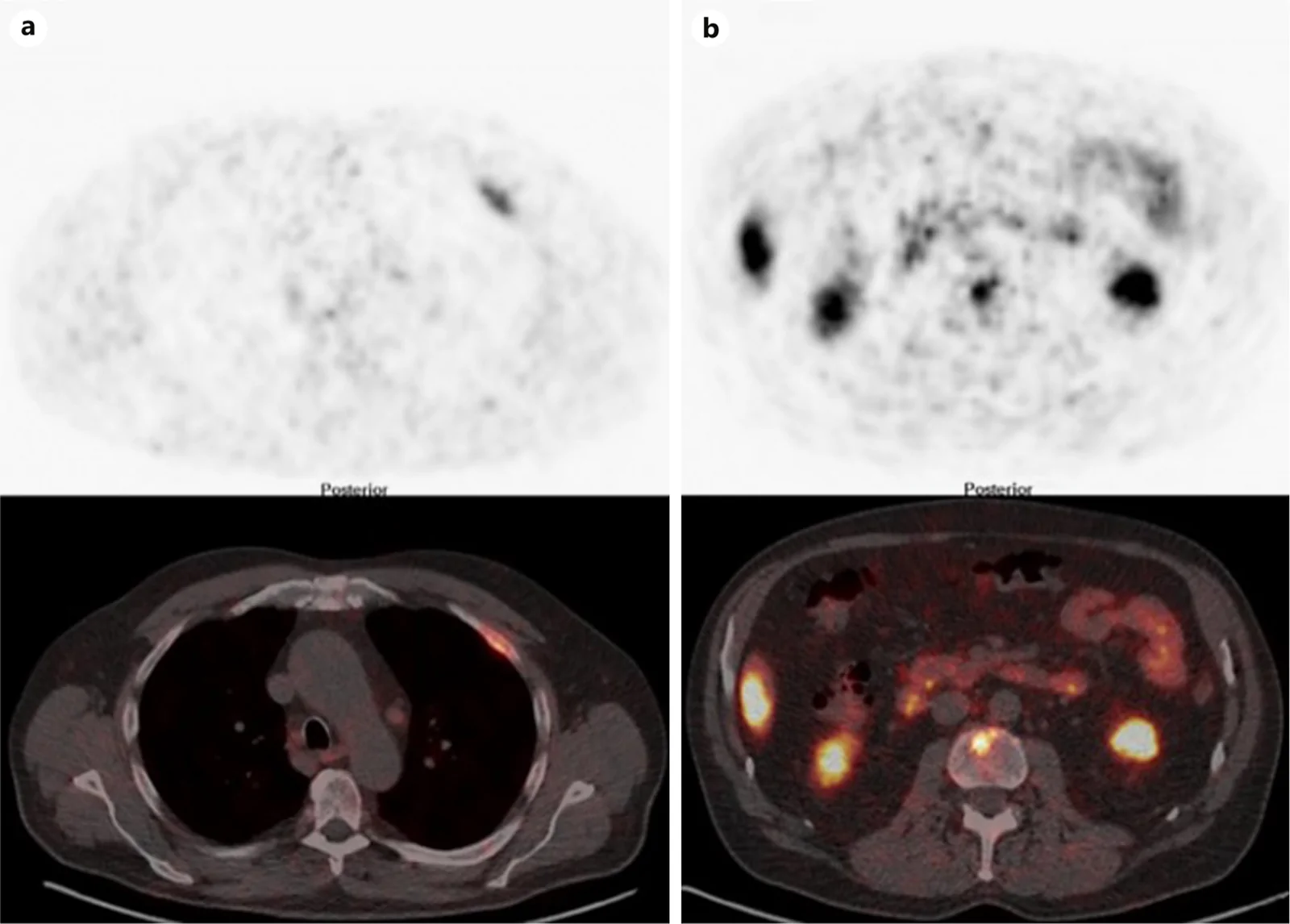
**a**, **b** 68Ga-DOTATATE-PET/CT of diffuse bone (**a**) and organ metastasis (**b**), December 2015.

In January 2016, octreotide therapy (30 mg every 4 weeks) was started, but despite the therapy, the 68Ga-DOTATATE-PET/CT performed in July 2016 confirmed the progression of the cancer (Fig. 6). At this point, the patient also had a diagnosis of a prostate cancer due to a high PSA level of 30. 65 ng/mL and a punch biopsy of the prostate. Octreotide therapy was discontinued at this time, and anti-androgen therapy was initiated. A neck sonography ordered in mid-February 2017 found a lymph node relapse (0.8 × 0.5 cm), and the calcitonin in the lesion after fine-needle puncture of the lymph node was 9.991 pg/mL. Therefore, another lymph node resection was performed in April 2017. Histological processing of the tissue sample revealed an MTC again. Basal calcitonin was now 141.7 pg/mL (Fig. 8 point C).

**Fig. 6.**
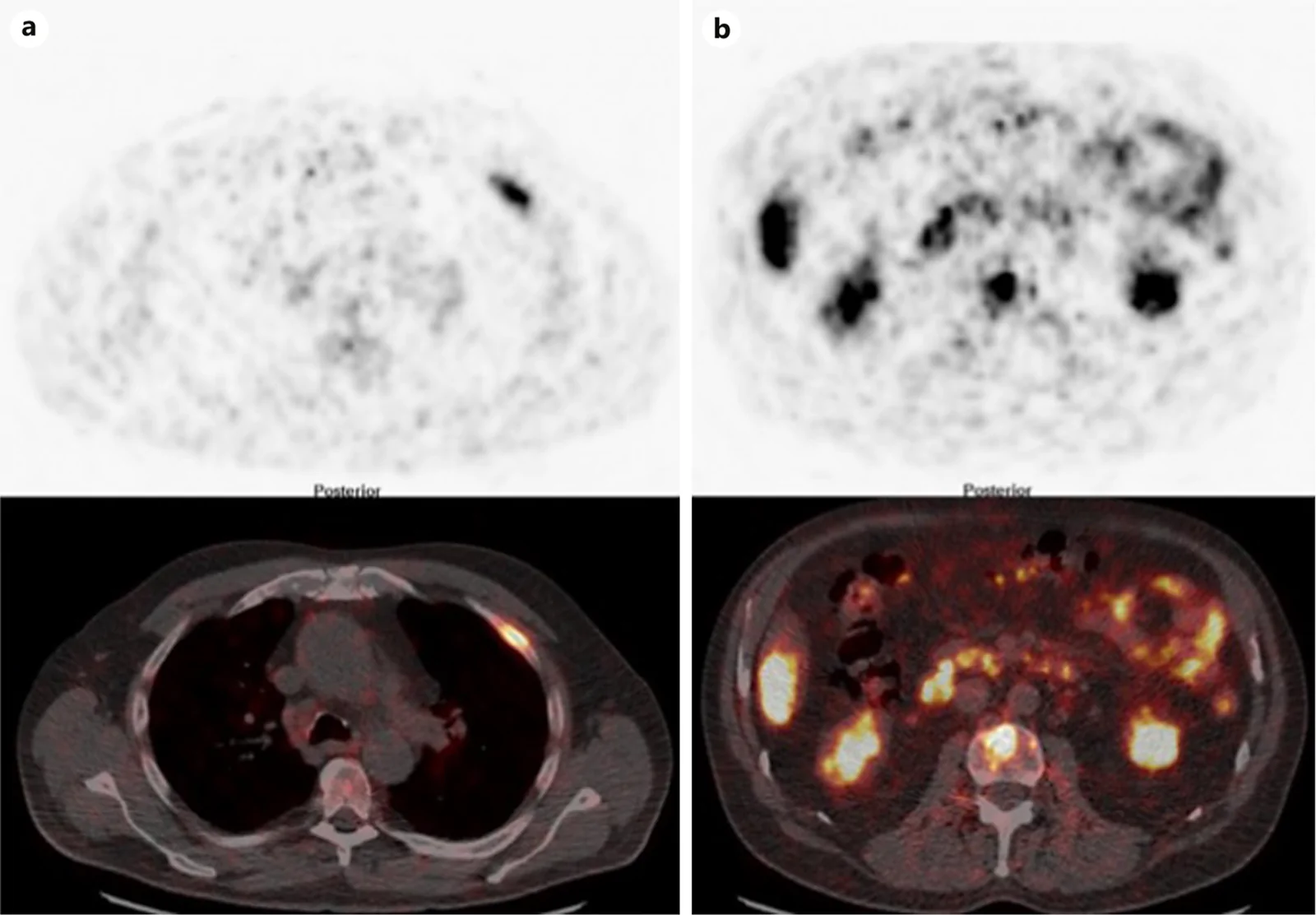
**a**, **b** 68Ga-DOTATATE-PET/CT shows a clear progressive disease, July 2016.

In May 2020, calcitonin showed a significant increase (657. 0 pg/mL) (Fig. 8 point E). Therefore, another [18F]-fluorodopa-PET/CT was performed and this time also a 68Ga-PSMA-PET/CT was performed (Fig. 7a, b). The latter showed significantly more bone and lymphatic lesions with a stronger tracer uptake than the [18F]-fluorodopa-PET/CT. In this, only a major metastasis in the third rib on the left with soft tissue component and a metastasis in the os ileum on the left were clearly visible, while the lesion in the 1st lumbar vertebrae was clearly positive only in the 68Ga-PSMA-PET/CT.

**Fig. 7.**
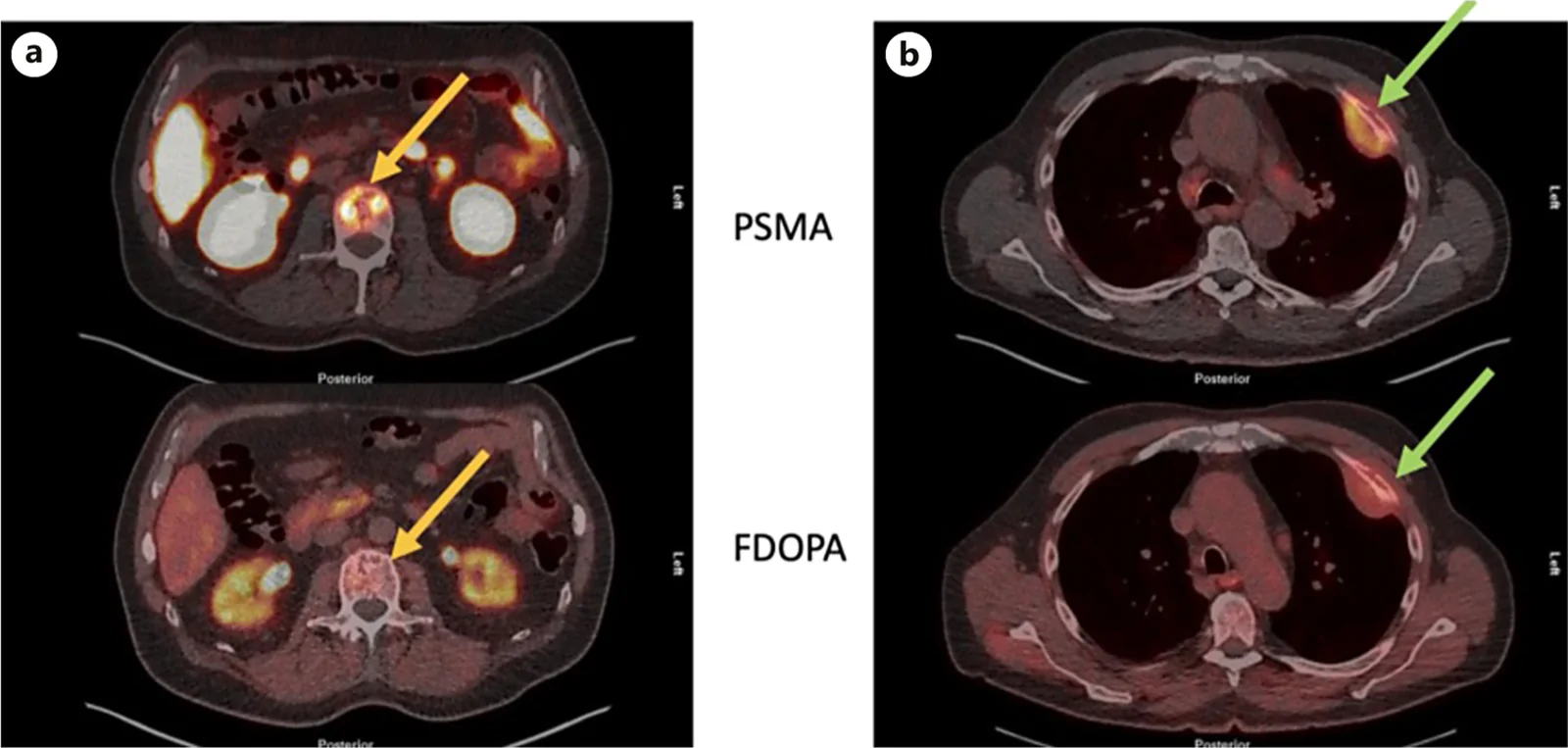
[18F]-Fluorodopa-PET/CT (**a**) and 68Ga-PSMA-PET/CT (**b**) were performed. The latter showed significantly more bone and lymphatic lesions with a stronger tracer uptake than the [18F]-fluorodopa-PET/CT.

The tumors were thus interpreted as prostate cancer metastases. Immediately thereafter, the patient suffered a pathological fracture of the 1st lumbar vertebrae, which was surgically stabilized. Surprisingly, histological processing of the surgical specimen revealed an osseous metastasis of the MTC. The rib lesion biopsy also revealed it to be an MTC metastasis.

Based on clinically manifested MTC metastases and calcitonin elevation to 954 pg/mL (Fig. 8 point F), the patient was enrolled in the selpercatinib Named Patient Program. Additional long-term diagnoses included atrial fibrillation and arterial hypertension under therapy.

#### Therapy with Selpercatinib

Selpercatinib (160 mg twice daily) was started in early August 2020. The patient responded quickly to this targeted therapy. There was a decrease in calcitonin levels after only 3 days. Within 14 days, the value had fallen significantly to 30.5 pg/mL (Fig. 8 point G). The patient developed a hypertension grade II, which was manageable by intensification of the antihypertensive medication.

In September 2020, the patient developed hypotension grade I, which resulted in a short-term dose reduction of selpercatinib to 80 mg twice daily for safety purposes. The calcitonin level increased in the short term to 49.4 ng/mL (Fig. 8 point H). The antihypertensive therapy was also reduced, and the patient’s condition stabilized within 2 weeks. He was able to receive the full dose of selpercatinib again. In October 2020, the calcitonin level fell again (27.2 ng/mL). The patient developed a mild proteinuria (grade I) and mild peripheral edema. His blood pressure was well controlled (RR: 135/90 mmHg).

At the follow-up visit in mid-May 2021, the patient showed a good general condition (ECOG 0-1). Calcitonin levels were low (18.8 ng/mL) and stable (Fig. 8 point I), and edema decreased under a diuretic therapy with furosemide retard (30 mg). The hypertension remained on grade I. The mild proteinuria (grade I) was stable. After 1 year of therapy, we performed a CT scan with which we observed stability in the dimensions of the tumor (RECIST 1.1: SD).

At the beginning of March 2022, the patient’s health status was stable (ECOG 0-1, calcitonin value: 20.4 pg/mL), the blood pressure normalized, and edema was resorbed under therapy. During this period, the patient developed a mild vertigo. Mild peripheral edema and dizziness are known as adverse reactions of selpercatinib. As proteinuria increased (grade II) during the visits in 2023, the dose of selpercatinib was reduced to 80 mg twice daily and so was maintained under control for the next visits.

At the last visit at the end of September 2025, the patient’s general condition and vertigo were unchanged. Calcitonin was stable low (17.8 ng/mL) (Fig. 8 point J), blood pressure at 135/75 mmHg, and proteinuria stabilized (grade I). Since the initiation of selpercatinib during the whole period of observation of 5 years, we observed a good tolerance of the medication and a progressive and sustained reduction of the tumor marker.

As of August 2025 (Fig. 8 point K), there is a sustained stable good serological partial remission of the neoplasia that has persisted until the point in time this case report was written, with acceptable tolerability of the treatment. The therapy is therefore currently being continued. The next clinical and laboratory chemical control examination is scheduled for December 2025.

## Discussion

In addition of the Data of the Phase 3 Trial of Julien Hadoux et al. [9] and new data added by the new publications of Hamidi et al. [10], in our observations we tested the real-world efficacy of targeted treatment with selpercatinib with manageable side-effect profile despite previous treatment and the long-term sustained remission along the 5-year therapy. We were able to confirm the long-lasting antineoplastic activity of selpercatinib in patients with RET-mutant MTC with mutations of RET, with and without previous treatment. As shown in the LIBRETTO-001 Clinical Trials [8] and again confirmed in Phase 3 LIBRETTO-531 ClinicalTrials [9], and the last real-world data of Hamidi et al. [10], the percentage of patients who had an objective response to selpercatinib was broadly consistent. During the trial, tumor regressions were observed in nearly all patients. Activity also appeared durable with 86% and 91% of responses ongoing at 12 and 24 months of therapy.

As consistent as our small number can be, the response we observed in our 2 patients in terms of reduction of the level of calcitonin was shown to be long lasting during the 5 years of observation and outdid the results by a reduction of more than 99% of the tumor marker in the patient 1 and more than 98% in the patient 2, suggesting a very good progression-free survival. Both of our patients were harboring the MEN2A mutation, suggesting a possible good outcome even in patients harboring a germline mutation as observed in other real-world comparisons [10].

Other tyrosine kinase inhibitors with a more broad effect as vandetanib or cabozantinib were previously approved for the treatment of metastatic MTC on the basis of phase 3 trials: cabozantinib and vandetanib [2, 3]. In the cabozantinib trial, the percentage of patients with an objective response was 28%, and the median progression-free survival was 11.2 months. In the vandetanib trial, the percentage of patients with an objective response was 45%, and the median progression-free survival was 30.5 months. Adverse events led to dose reduction in 35% of the patients receiving vandetanib and 79% of those receiving cabozantinib and permanent discontinuation of therapy in 12% and 16% of the patients, respectively.

By comparison, the selectivity of selpercatinib for RET leads to few and lower grade adverse events. Even in our observation, the few adverse events our patients experienced were of grade 1 and 2 and the only dose reductions were reversible after only a few months and we observed a persistent good tolerability of the drug.

In both of our patients, stereotactic radiation was not used as a possible therapy. As proved from Anastasios Maniakas et al. [11], the use of radiotherapy has drastically decreased in the treatment of metastatic MTC and its indications have become more selective in neoplasia harboring an RET mutation, where a targeted therapy offers better results in terms of PFS and OS. Some real-world data suggest that the use of targeted RET therapy could be an asset even in the neoadjuvant treatment of patients with locally advanced MTC [12].

## Conclusions

- In patients with MTC, genetic testing for RET mutations is absolutely indicated. As it can be used to identify an MEN2A mutation in the family.
- Calcitonin is a reliable biomarker of progression and follow-ups in MTC.
- Unclear thyroidal lesions should always be examined histologically.
- Selpercatinib proved to be a very good antineoplastic drug for patients with RET-mutated, advanced, and inoperable MTC, not just in terms of control of the neoplasia, but even in terms of potential side effects.
- In these 2 MTC patients, the antineoplastic treatment with selpercatinib was rapidly effective and well tolerated with minor adverse events. Similar efficacy was achieved despite reduced selpercatinib doses.
- Therefore, this good and now available first-line treatment option for RET-mutated advanced MTC can and should be used without hesitation.

To study the actual improvement in progression-free survival in those patients and the possible uses of selpercatinib in other RET-mutated cancers, even in neoadjuvant settings, longer follow-up periods and international collaborations are needed.

## Statement of Ethics

This case report was conducted in accordance with the principles of the Declaration of Helsinki. Written informed consent was obtained from both patients for the publication of their clinical data and any accompanying images. Institutional Review Board approval was not required for this study, in accordance with local regulations for case reports. This case report was prepared in accordance with the CARE guidelines. The completed CARE checklist has been submitted as online supplementary material (for all online suppl. material, see https://doi.org/10.1159/cro/abzag009).

## Conflict of Interest Statement

The authors declare that the research was conducted in the absence of any commercial or financial relationships that could be construed as a potential conflict of interest.

## Funding Sources

This research did not receive any specific grant from funding agencies in the public, commercial, or not-for-profit sectors.

## Author Contributions

Dr. Luciano Severgnini and Dr. Bernd Hartmann were responsible for patient management and data collection. Dr. Luciano Severgnini performed the literature review and drafted the manuscript. Doz. Thomas Winder and Dr. Bernd Hartmann critically revised the manuscript for important intellectual content. All authors contributed to the article and approved the submitted version.

## Data Availability

Due to the sensitive nature of clinical data, additional datasets are not publicly available but may be provided by the corresponding author upon reasonable request.

## References

[1] Ciampi R , Romei C, Ramone T, Prete A, Tacito A, Cappagli V, . Genetic landscape of somatic mutations in a large cohort of sporadic medullary thyroid carcinomas studied by next-generation targeted sequencing. 2019;20:324–336. 10.1016/j.isci.2019.09.030 PMC6817656 31605946

[2] Wells SA Jr , Robinson BG, Gagel RF, Dralle H, Fagin JA, Santoro M, . Vandetanib in patients with locally advanced or metastatic medullary thyroid cancer: a randomized, double-blind phase III trial. J Clin Oncol. 2012;30(2):134–41. 10.1200/JCO.2011.35.5040 PMC3675689 22025146

[3] Elisei R , Schlumberger MJ, Müller SP, Schöffski P, Brose MS, Shah MH, . Cabozantinib in progressive medullary thyroid cancer. J Clin Oncol. 2013;31(29):3639–46. 10.1200/JCO.2012.48.4659 PMC4164813 24002501

[4] Subbiah V , Velcheti V, Tuch BB, Ebata K, Busaidy NL, Cabanillas ME, . Selective RET kinase inhibition for patients with RET-altered cancers. Ann Oncol. 2018;29(8):1869–76. 10.1093/annonc/mdy137 PMC6096733 29912274

[5] Subbiah V , Gainor JF, Rahal R, Brubaker JD, Kim JL, Maynard M, . Precision targeted therapy with BLU-667 for RET-driven cancers. Cancer Discov. 2018;8(7):836–49. 10.1158/2159-8290.CD-18-0338 29657135

[6] Subbiah V , Cote GJ. Advances in targeting RET-dependent cancers. Cancer Discov. 2020;10(4):498–505. 10.1158/2159-8290.CD-19-1116 PMC7125013 32094155

[7] Subbiah V , Yang D, Velcheti V, Drilon A, Meric-Bernstam F. State-of-the-art strategies for targeting RET-dependent cancers. J Clin Oncol. 2020;38(11):1209–21. 10.1200/JCO.19.02551 PMC7145587 32083997

[8] Lori J . Wirth, MD, Eric Sherman, MD, Bruce Robinson, MD, . Efficacy of selpercatinib in RET-altered thyroid cancers. 10.1056/NEJMoa2005651 PMC10777663 32846061

[9] Hadoux J , Elisei R, Brose MS, Hoff AO, Robinson BG, Gao M, . Phase 3 Trial of Selpercatinib in Advanced RET-Mutant Medullary Thyroid Cancer. N Engl J Med. 2023;389:1851–18. 10.1056/NEJMoa2309719 37870969

[10] Hamidi S , Yedururi S, Hu MI, Busaidy NL, Sherman SI, Jimenez C, . +9 efficacy and safety of selective RET inhibitors in patients with advanced hereditary medullary thyroid carcinoma. Thyroid. 2025;35(1):6–17. 10.1089/thy.2024.0495 39630530

[11] Maniakas A , Sullivan A, Hu MI, Busaidy NL, Cabanillas ME, Dadu R, . +18 decreasing utilization for postoperative radiation therapy in locoregionally advanced medullary thyroid cancer. Head Neck. 2024;46(2):328–35. 10.1002/hed.27584 38009416

[12] Contrera KJ , Gule-Monroe MK, Hu MI, Cabanillas ME, Busaidy NL, Dadu R, . +14 neoadjuvant selective RET inhibitor for medullary thyroid cancer: a case series. Thyroid. 2023;33(1):129–32. 10.1089/thy.2022.0506 36503246

